# Droplet Imbibition into Paper Coating Layer: Pore-Network Modeling Simulation

**DOI:** 10.1007/s11242-018-1116-0

**Published:** 2018-07-13

**Authors:** X. Yin, H. Aslannejad, E. T. de Vries, A. Raoof, S. M. Hassanizadeh

**Affiliations:** Department of Earth Sciences, Utrecht University, Utrecht, The Netherlands

**Keywords:** Paper coating layer, Imbibition, Pore-network model, Droplet penetration

## Abstract

Liquid penetration into thin porous media such as paper is often simulated using continuum-scale single-phase Darcy’s law. The underlying assumption was that a sharp invasion front percolates through the layer. To explore this ambiguous assumption and to understand the controlling pore-scale mechanisms, we have developed a dynamic pore-network model to simulate imbibition of a wetting phase from a droplet into a paper coating layer. The realistic pore structures are obtained using the FIB-SEM imaging of the coating material with a minimum resolution of 3.5 nm. Pore network was extracted from FIB-SEM images using Avizo software. Data of extracted pore network are used for statistically generating pore network. Droplet sizes are chosen in the range of those applicable in inkjet printing. Our simulations show no sharp invasion front exists and there is the presence of residual non-wetting phase. In addition, penetration of different sizes of droplets of different material properties into the pore network with different pore body and pore throat sizes are performed. We have found an approximately linear decrease in droplet volume with time. This contradicts the expected $\sqrt{t}$-behavior in vertical imbibition that is obtained using macroscopic single-phase Darcy’s law. With increase in flow rate, transition of imbibition invasion front from percolation-like pattern to a more sharper one with less trapping of non-wetting phase is also reported. Our simulations suggest that the single-phase Darcy’s law does not adequately describe liquid penetration into materials such as paper coating layer. Instead Richards equation would be a better choice.

## Introduction

The spreading and penetration of liquid droplets into a porous layer is ubiquitous in several industrial applications as well as natural systems. An important industrial example is the penetration of ink droplets into paper during inkjet printing. While viscous and capillary effects are the main transport mechanisms, many other phenomena such as evaporation, solidification, adsorption and diffusion affects the process. Research has been done to investigate transport mechanisms from experimental, theoretical, and/or numerical points of view. Reviews on this topic can be found in Gambaryan-Roisman (2014), Daniel and Berg (2006) and Kettle et al. (2010).

For the study of spreading on a porous layer, much attention has been given to the droplet shape and size evolution, especially the physics taking place in the vicinity of the droplet edges (Starov et al. 2002, 2003; Davis and Hocking 2000; Clarke et al. 2002; Anderson 2005; Alleborn and Raszillier 2004). Droplet-air interface is usually assumed to have a spherical or parabolic shape or it is modeled by the lubrication theory. Fluid behavior inside the porous medium is then interpreted based on the droplet spreading information. In these studies, single-phase Darcy’s law is often employed to model penetration inside porous media with the underlying assumption of a sharp wetting front. For one-dimensional flow, this leads to a traveling sharp front which propagates proportional to $\sqrt{t}$. For example, Lembach et al. (2011) visualized the transient spreading and infiltration of a droplet into a bed of transparent glass beads. Penetration depth and the information of the width of the front were obtained using image analysis. They employed single-phase Darcy’s law to interpret their experimental measurements. A capillary pressure was assumed to act at the traveling wet/dry sharp interface, and they addressed the important effect of dynamic contact angle assuming it to depend on the front velocity. However, the use of single-phase Darcy’s law for description of droplet penetration into porous medium may be questioned as the underlying assumption of sharp wetting front may not always hold (Geiger and Durnford 2000; Markicevic and Navaz 2010; Gambaryan-Roisman 2014).

Pore-scale simulation offers another approach to explore this process. Frank and Perré (2012) employed Lattice Boltzmaan Method (LBM) to simulate droplet spreading over a hypothetical porous medium made of parallel vertical pores. Their numerical results showed power-law evolution of the wetted zone radius over time. They also discussed the interplay between lateral spreading and capillary penetration. Meng et al. (2014) used Smoothed Particle Hydrodynamics(SPH) method to investigate main factors affecting the spreading process on a porous substrate including the contact angle, pore sizes and the gravity effects. The porous substrate is represented by a solid cube with square columns longitudinally penetrating the whole depth. Direct simulations in these studies often employ ideal simplified geometries and topology and dynamic contact angle and contact line pinning were not appropriately resolved .

A large body of works [see review by Joekar-Niasar and Hassanizadeh (2012)] exist for simulation of imbibition in porous media using pore-network models (Dias and Payatakes 1986a, b; Blunt 2001; Blunt and Scher 1995; Constantinides and Payatakes 1996; Hughes and Blunt 2000; Mogensen and Stenby 1998; Nguyen et al. 2006). These algorithms could suffer one of the followings: (1) network with regular geometry/topology; (2) failure to include corner flow/film flow; (3) ignorance of capillary pressure at pores; (4) rule-based description of interface morphology; (5) rule-based dynamic algorithm.

In two-pressure algorithm, both pore body and pore throat can be filled with two fluids, each fluid with its own pressure. Interface position or shape is included in pore body capillary pressure-saturation relationship. Pressure, flux and saturation are calculated separately for each fluid. This algorithm was initially developed by Thompson (2002) to study imbibition process in fibrous materials. Later this algorithm was improved and implemented onto structured network with fixed coordination number of six to study theories of two-phase flow in porous media (Joekar-Niasar et al. 2010; Joekar-Niasar and Hassanizadeh 2011a, b) and wicking behavior (Joekar-Niasar and Hassanizadeh 2012a). For two-phase flow involving two fluids with significant different viscosities, like water-air system, pressure drop in less viscous fluid may be negligible, and the above two-pressure algorithm may be simplified accordingly.

In this work, we have developed a dynamic single-pressure pore-network model to study the infiltration of a liquid into paper. Our model simulates the complex geometry and topology of the coating layer by constructing a network based on the statistical information obtained from a previous study using FIB-SEM (Scanning Electron Microscopy combined with Focused Ion Beam) imaging of a coating layer (Aslannejad et al. 2017). At this stage of the study, we only simulate the penetration of liquid inside the coating layer and the droplet evolution outside of the porous medium is not modeled, but it is considered as a known boundary condition.

The objectives of our study are as follows:

1. To develop a dynamic imbibition pore-network model and simulate a liquid penetration into the paper coating layer.
2. To study imbibition rate of different sizes of droplets and to explore the effects of the porous material properties and pore size.
3. To investigate displacement pattern during penetration and explore whether the sharp front assumption for liquid penetration is valid.

Paper coating layer properties are introduced in Sect. 2. Details of the pore-network model are described in Sect. 3. Simulation results are shown and discussed in Sect. 4. Finally, the conclusions are provided in Sect. 5.

## Properties of Paper Coating Layer

### Properties Based on FIB-SEM Image

**Fig. 1:**
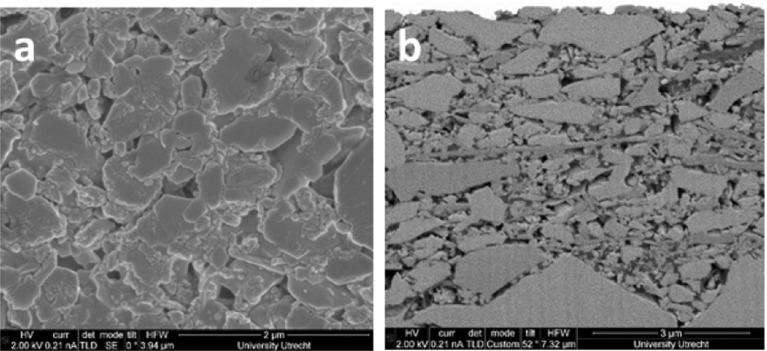
**a** Surface view of the paper coating; **b** cross-sectional view of paper coating From Aslannejad et al. (2017)

Figure 1 gives the surface and a vertical cross section of the coating layer from FIB-SEM imaging. Resolution in cross section is 3.5 nm and each slice has thickness of 25 nm.

Porosity of paper coating layer is about 0.34 and permeability is found to be 0.1 m Darcy, obtained by solving steady-state Stokes equations using Geodict(Math2Market, Kaiserslautern, Germany). More details can be found in Aslannejad et al. (2017).

**Fig. 2:**
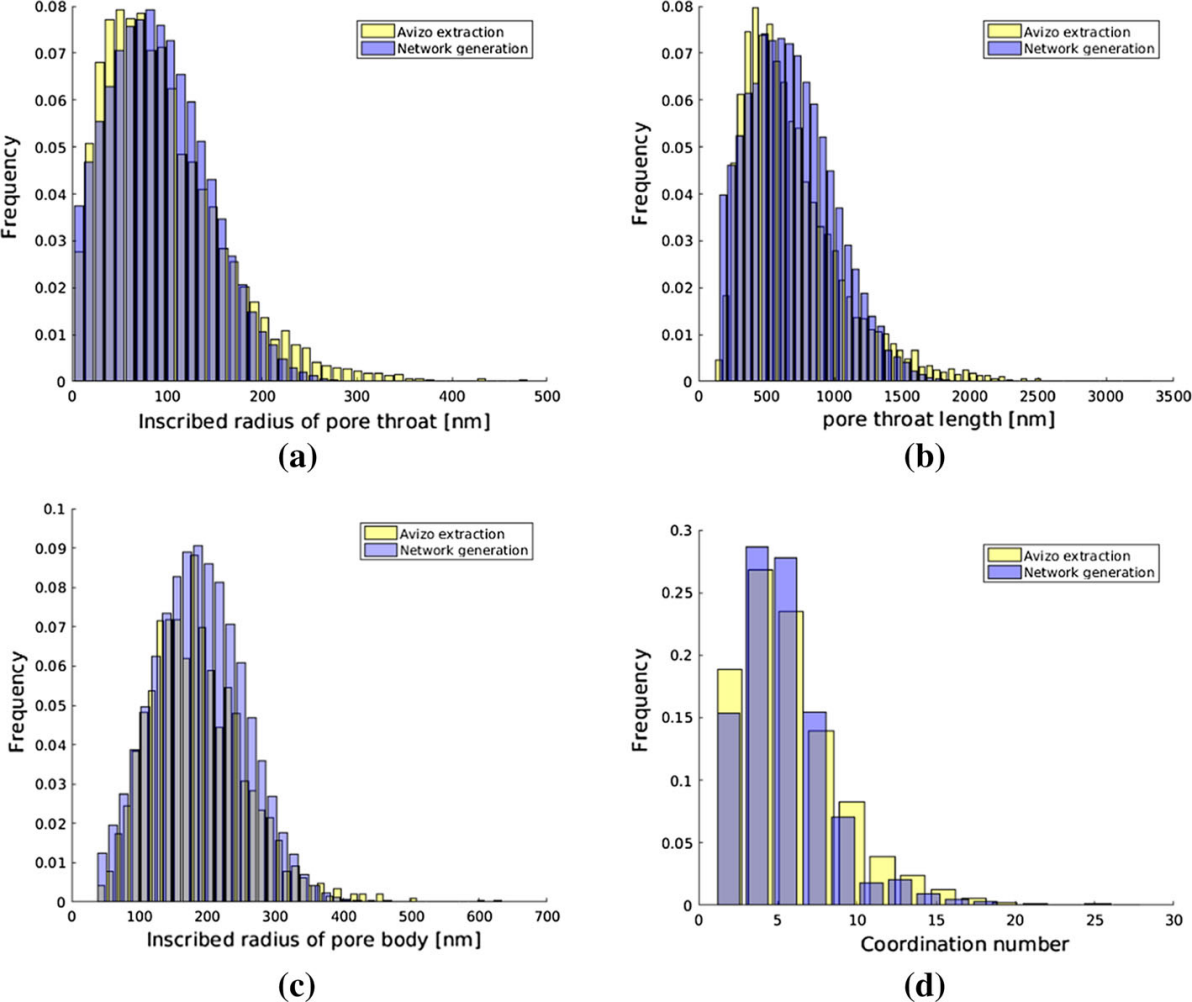
Size distributions of **a** inscribed radii of pore throats; **b** pore throat length; **c** inscribed radii of pore bodies; **d** coordination number; of Avizo extracted network and generated network

Pore-network structure was extracted from FIB-SEM images using Avizo software (FEI Visualization Sciences Group). Figure 2 provides the size distribution of inscribed radii of pore throats, inscribed radii of pore bodies, length of pore throats and coordination number. These data are used later in Sect. 3 to construct the pore network.

## Pore-Network Model Description

A single-pressure algorithm is developed for the simulation of imbibition process, where a viscous liquid is the wetting phase and air is the non-wetting phase. Wetting phase pressure will be calculated while the air phase is assumed to be at a constant and uniform pressure at all times. This is a valid assumption given the negligible viscosity of the air phase.

### Model Features

*Structure and geometry* Due to big difference in droplet sizes and pore sizes, a droplet over paper coating layer can cover quite many pores. The window in high resolution FIB-SEM imaging is not big enough for it. So, the pore network is generated statistically using an irregular network generator developed by Raoof and Hassanizadeh (2012). Sizes distribution and coordination number distribution of generated pore network and extracted pore network are shown in Fig. 2.

Pore bodies are considered to have a cubic shape and pore throats are assigned square cross sections. Porosity of the network is 0.37, close to that of 0.34 of the sample. Intrinsic permeability of the generated pore network is found to be 0.12 m Darcy, which compares well with 0.1 m Darcy obtained using image-based direct simulations. The reasonable match between the parameters implies that the extracted pore structures are representative of the coating layer.

*Assumptions* Assumptions employed in the pore-network algorithm and modeling are as follows:

1. The volume of pore throats are assumed to be negligible compared to the volume of pore bodies, as a common assumption in pore-network modeling. This implies that the filling of a pore throat is assumed to occur in no time.
2. No hydraulic resistance is assigned to pore bodies; their resistance to the flow is assumed to be negligible compared to that of pore throats.
3. Non-wetting phase (i.e., the air phase), which has a much lower viscosity value relative to the wetting fluid, is assumed to be at a constant and uniform pressure distribution.
4. No gravity effects are included due to the small size of the computational domain and droplet.
5. Flow of wetting phase through corners of pore elements is taken into account. Therefore, any pore body or pore throat can be simultaneously occupied by both wetting phase and non-wetting phase.
6. As coupling between droplets spreading and penetration onto paper coating layer is complex, we only consider the penetration part. Namely, a droplet penetrates into paper coating layer through a circle with the diameter equal to that of the droplet. Pressure of droplet is assumed the same as that of atmosphere pressure.

*System parameters* Table 1 provides fluid properties used in the simulations. Contact angle value 45 degree is taken from Järnström et al. (2010). They determined contact angle based on topography from nanometer to millimeter length scales by atomic force microscopy (AFM), confocal optical microscopy (COM), and X-ray tomography. Zero degree contact angle is also used to study influence of contact angle. Wetting phase viscosity of 0.040 kg $\hbox {m}^{-1}\,\hbox {s}^{-1}$ corresponds to a glycerol solution used in experiments by Clarke et al. (2002). Three sets (M1, M2, M3) of material properties are adopted.

To explore effects of pore body and pore throat sizes on imbibition, we simulate two different scenarios: (1) G1: based on sizes distributions shown in Fig. 2; (2) G2: increasing pore body and pore throat sizes by a factor of 2, keeping coordination number distribution unchanged.

**Table 1:** Material properties sets

Sets no.	Contact angle	Interfacial tension	Wetting fluid viscosity
	$\theta$ ($^\circ$)	$\sigma ^{wn}$ (kg $\hbox {s}^{-2}$)	${\mu }^{w}$ (kg $\hbox {m}^{-1}\,\hbox {s}^{-1}$)
M1	45	0.072	0.001
M2	45	0.038	0.040
M3	0	0.038	0.040

### Solution Algorithm

Local ( i.e., the pore level) capillary pressure for a given pore body *i* is defined as:

1 $$\begin{aligned} p_{i}^{c}=p_{i}^{n}-p_{i}^{w}=p_{i}^{c}(s_{i}^{w}) \end{aligned}$$

where $p_{i}^{c}$ is capillary pressure, $p_{i}^{\alpha }$ and $s_{i}^{\alpha }$ denote pressure and saturation of $\alpha$ phase in pore body *i*. $p_{i}^{c}(s_{i}^{w})$ relationship is given in Sect. 3.3.

Initially, all pore bodies are assumed to have a minimum wetting phase saturation (we set $s_{min}^{w}=0.005$) except the first row of pore bodies in contact with the droplet which remain saturated. Those boundary pore bodies are assumed to be fully saturated. Thus, initially all internal pore bodies have a large negative wetting phase pressure given by $p_{i}^{c}(s_{i}^{w})$ relationship (we have imposed a maximum of $p_{i}^{c}$ of $10^{6}$ Pa ). Saturated boundary pores are always at atmospheric pressure until the droplet fully penetrates into the layer, at which time the simulation is ended.

The droplet infiltration starts by the wetting phase flowing from the saturated boundary pores into the internal pores. Their saturation, and thus their pressure rise and, subsequently, wetting phase can flow into the neighboring pores. The wetting phase flow occurs via pore throats and its rate can be calculated using Hagen–Poisseulle formula:

2 $$\begin{aligned} q_{ij}^{w}=K_{ij}^{w}(p_{i}^{w}-p_{j}^{w}) \end{aligned}$$

where *i* is the upstream node. Here, $K_{ij}^{w}$ is the conductivity of wetting phase in the pore throat, for which a relationship is given later in Sect. 3.3.

The saturation update for each pore body is made based on the following volume balance equation:

3 $$\begin{aligned} {{V}_{i}}\frac{\Delta s_{i}^{w}}{\Delta t}=-\sum \limits _{j=1}^{{{N}_{i}}}{K_{ij}^{w}(p_{i}^{w}-p_{j}^{w})}=-q_{i}^{w} \end{aligned}$$

where $V_i$ is volume of pore body *i*, ${N}_{i}$ is coordination number of pore body *i* and $q_{i}^{w}$ is total flux of pore body *i*. The method for determining time step $\Delta t$ will be explained shortly below. The calculation is done fully explicitly. Note that the saturation update needs to be done for active unsaturated pore bodies, those for which at least one pore throat has been invaded by wetting phase. As long as no internal pore body has become fully saturated with the wetting phase, the updating of pore body saturation and pressure, invasion of new pore throats and flow calculation continues. However, at each step, we check whether a given pore throat can be invaded by the wetting phase, following the criteria discussed in Sect. 3.3.

As soon as one or more internal pore bodies become fully saturated with the wetting phase, then we have to solve for the pressure of those pore bodies based on the following volume balance equation:

4 $$\begin{aligned} \sum \limits _{j=1}^{{{N}_{i}}}{K_{ij}^{w}(p_{i}^{w}-p_{j}^{w})=0} \end{aligned}$$

Here $K_{ij}^{w}$ is calculated explicitly. The domain of saturated pore bodies is surrounded by unsaturated pore bodies. The pressure of unsaturated pore bodies (known from the current time step) is used as boundary condition values for the domain of saturated pore bodies. Once the pressure of all saturated pore bodies are calculated, steps described above for updating saturation of unsaturated pore bodies will be repeated.

**Fig. 3:**
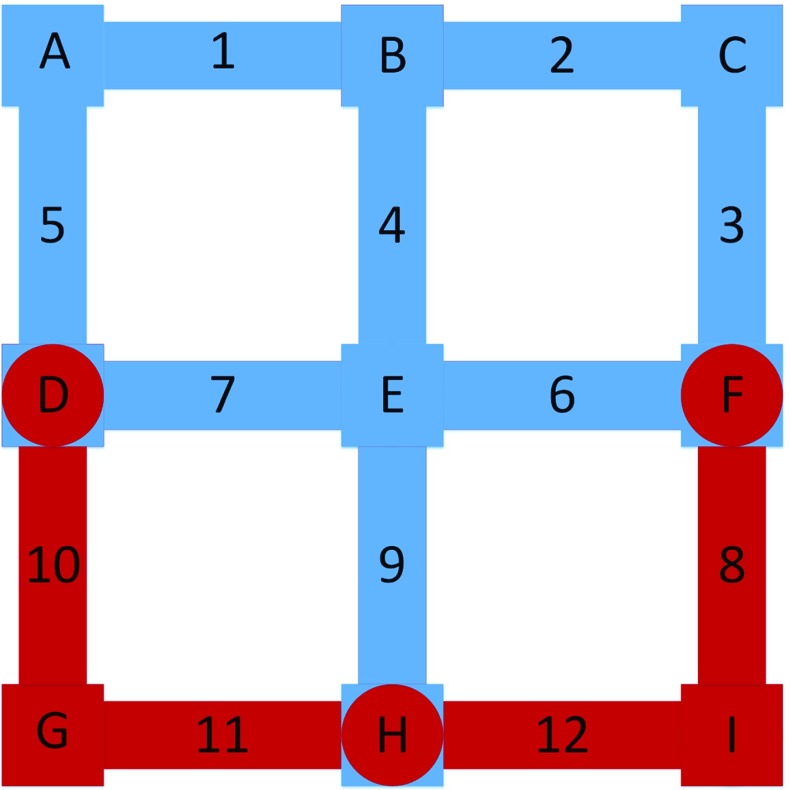
Schematic diagram of boundary pores, internal unsaturated pores and saturated pores

Figure 3 gives schematic diagram of part of the domain with boundary pores and internal saturated and unsaturated pores, where blue color shows the wetting phase and red color shows the non-wetting phase. In this figure, pore bodies are denoted from A to I and pore throats are denoted from 1 to 12. No initial minimum saturation is shown in the schematic figure and no attempt is made to represent the real interface shape. A, B, C are boundary pore bodies and pore bodies {D,E,F,H} have been invaded by the wetting phase. Depending on invasion criteria, pore throats {3,4,5,6,7,9} are invaded and fully occupied by the wetting phase while pore throats {8,10,11,12} are not invaded yet.

*Time step* During imbibition, time step $\Delta t$ is taken to be equal to the smallest filling time $\Delta {{t}_{i}}$ of all active unsaturated pore bodies, but it is possible to have local drainage in some pore bodies. Allowing pore bodies to be infiltrated to $s_{i}^{w}=1$ or drained to $s_{i}^{w}=0$. $\Delta {{t}_{i}}$ is determined as:

5 $$\begin{aligned} \Delta {{t}_{i}}={\left\{ \begin{array}{ll} \frac{{{V}_{i}}}{q_{i}^{w}}(s_{i}^{w})&{} q_{i}^{w}>0 \\ \frac{{{V}_{i}}}{q_{i}^{w}}(1-s_{i}^{w})&{} q_{i}^{w}<0 \end{array}\right. } \end{aligned}$$

A global time step is then selected as the minimum time step of all pore bodies:

6 $$\begin{aligned} \Delta t=\min \{\Delta {{t}_{i}}\} \end{aligned}$$

A saturation truncation value of $10^{-3}$ is adopted to ensure finite time step. When local saturation in a pore body is close to the target saturation values (1 or 0) within the truncation value, that pore body will not be included in global time step determination.

### Local Rules

*Capillary pressure for pore bodies* Assuming that the wetting phase resides symmetrically in all eight corners of pore bodies, capillary pressure-saturation relationship for cubic pore bodies can be as (Joekar-Niasar et al. 2010):

7 $$\begin{aligned} p_{i}^{c}(s_{i}^{w})=\frac{2{{\sigma }^{wn}}cos \theta }{{{R}_{i}}(1-\exp (-6.83s_{i}^{w}))} \end{aligned}$$

where ${R}_{i}$ is the radius of inscribed sphere of pore body *i*. Here, we have not considered the possibility for different interface shapes in a pore body due to different filling states of its neighbors (Lenormand and Zarcone 1984).

*Entry capillary pressure for pore throats* Entry capillary pressure for a square pore throat is defined as (Mason and Morrow 1991; Mayer and Stowe 1965; Princen 1970):

8 $$\begin{aligned} p_{ij}^{en}=\frac{{{\sigma }^{wn}}}{{{r}_{ij}}}\left( \frac{\theta +{{\cos }^{2}}\theta -\pi /4-\sin \theta \cos \theta }{\cos \theta -\sqrt{\pi /4-\theta +\sin \theta \cos \theta }}\right) \end{aligned}$$

where ${r}_{ij}$ is the radius of inscribed circle of pore throat cross section.

Based on entry capillary pressure, we determine how will wetting phase displace non-wetting phase, via corner flow or through main terminal meniscus. For high capillary pressure, wetting phase can only invade non-wetting phase via corner flow and two phases co-exist within a single pore cross section; when the capillary pressure decreases, to become equal to the entry capillary pressure, wetting phase will occupy the whole pore cross section. Then, for corner flow case, we check whether snap-off will happen based on snap-off criteria described by Eq. (9).

*Invasion criteria and trapping* From one time step to another, a pore throat will be assumed to get invaded by the wetting phase only if at least one of its neighboring pore bodies has reached a wetting phase saturation of 0.477 (corresponding to the case where the non-wetting phase filling the inscribed sphere of the pore body). At first, only corners are assumed to become filled. The radius of the meniscus formed in the corner depends on the wetting phase pressure, and is given by Eq. (13). Capillary pressure of pore throat is set the same as its neighboring upstream pore body capillary pressure.

If the wetting phase pressure in one of the neighboring pore bodies is high enough, the whole pore cross section will be invaded by the wetting phase. So the criteria for the full invasion of a pore throat is $p_{ij}^{c} < p_{ij}^{en}$ by the wetting phase in the unsaturated neighboring pore body. For wetting phase saturated pore body, wetting phase will invade the narrower pore throat through main terminal meniscus. However, even if one or both invasion criteria are met, a pore throat will not be invaded if it is considered to be trapped (Al-Futaisi and Patzek 2003). Details about different scenarios of invasion and trapping are provided in “Appendix”.

In general, when a pore throat is not considered to be trapped, it can always be invaded by the wetting phase (independent of the applied boundary pressure) as corner flow can always occur. However, we have imposed the requirement that the saturation of one of the neighboring pore bodies must exceed 0.477, to have accessibility to the entry of the pore throat which is to be invaded.

*Snap-off* During imbibition, snap-off may happen in a pore throat when capillary pressure in the pore throat drops below a threshold value so that stable corner interface is not supported any more. For square cross-sectioned pore throat, ignoring dynamic contact angle effects, the criterion on snap-off is defined as (Vidales et al. 1998):

9 $$\begin{aligned} p_{ij}^{c} \le \frac{{{\sigma }^{wn}}}{{{r}_{ij}}}(\cos \theta -\sin \theta ) \end{aligned}$$

Once snap-off happens in a pore throat, it will be fully occupied by the wetting phase and its conductivity will be changed accordingly.

*Conductivities of pore throats* As we only solve for the wetting phase pressure, only wetting phase conductivity will be needed in the simulations. The conductivity of the wetting phase of pore throats depends on fluids occupancy in the pore throat cross section. During imbibition, the following states are possible:

1. Pore throat is fully occupied by wetting phase. Then the conductivity is given by (Azzam and Dullien 1977).
  10 $$\begin{aligned} K_{ij}^{w}=\frac{\pi }{8{{\mu }^{w}}{{l}_{ij}}}r{{_{ij}^\mathrm{eff}}^{4}} \end{aligned}$$
  where ${l}_{ij}$ is length of pore throat and
  11 $$\begin{aligned} r_{ij}^\mathrm{eff}=\sqrt{\frac{\pi }{4}}{{r}_{ij}} \end{aligned}$$
2. Pore throat is occupied by the wetting phase in the corner while non-wetting phase is in the middle (Ransohoff and Radke 1988).
  12 $$\begin{aligned}&K_{ij}^{w}=\frac{4-\pi }{\beta {{\mu }^{w}}{{l}_{ij}}}r{{_{ij}^{c}}^{4}} \end{aligned}$$
  13 $$\begin{aligned}&r_{ij}^{c}=\frac{{{\sigma }^{wn}}}{p_{ij}^{c}}\left( \frac{\theta +{{\cos }^{2}}\theta -\pi /4-\sin \theta \cos \theta }{\cos \theta -\sqrt{\pi /4-\theta +\sin \theta \cos \theta }}\right) \end{aligned}$$
  Dimensionless resistance $\beta$ depends on pore throat cross section shape, geometry of wetting/non-wetting interface and proper boundary condition at interface (Zhou et al. 1997). In this study, constant value of 100 has been chosen. Due to suppressed snap-off (see Sect. 4), we think this will not influence much over our simulation results.

### Computational Procedure

*Computational procedure* The procedure for dynamic primary imbibition simulation is:

1. Set boundary condition and initial condition for the pore network;
2. Determine conductivity of pore throats based on fluid occupancy as well as trapping and invasion criteria;
3. Solve wetting phase volume balance equations for the saturated pore bodies and calculate the pressure field;
4. Calculate fluxes based on the conductivity values determined in step 2, then determine time step;
5. Update saturation and pressure of unsaturated active pore bodies;
6. Continue with step 2 and repeat the process.

## Dynamic Simulation Results and Discussion

In inkjet system, the droplet volume typically ranges from 4 to 18 *pL* ($pL=picoliter$) with minimum droplet size of 2 *pL* (Clarke et al. 2002; Kettle et al. 2010). Therefore, we simulate for different droplet sizes of 2, 4, 8, 12 *pL*, penetrating into two different pore networks (G1, G2) with material properties (M1, M2, M3), described in Sect. 3.1.

### Symmetry of Saturation Pattern During Droplet Penetration

**Fig. 4:**
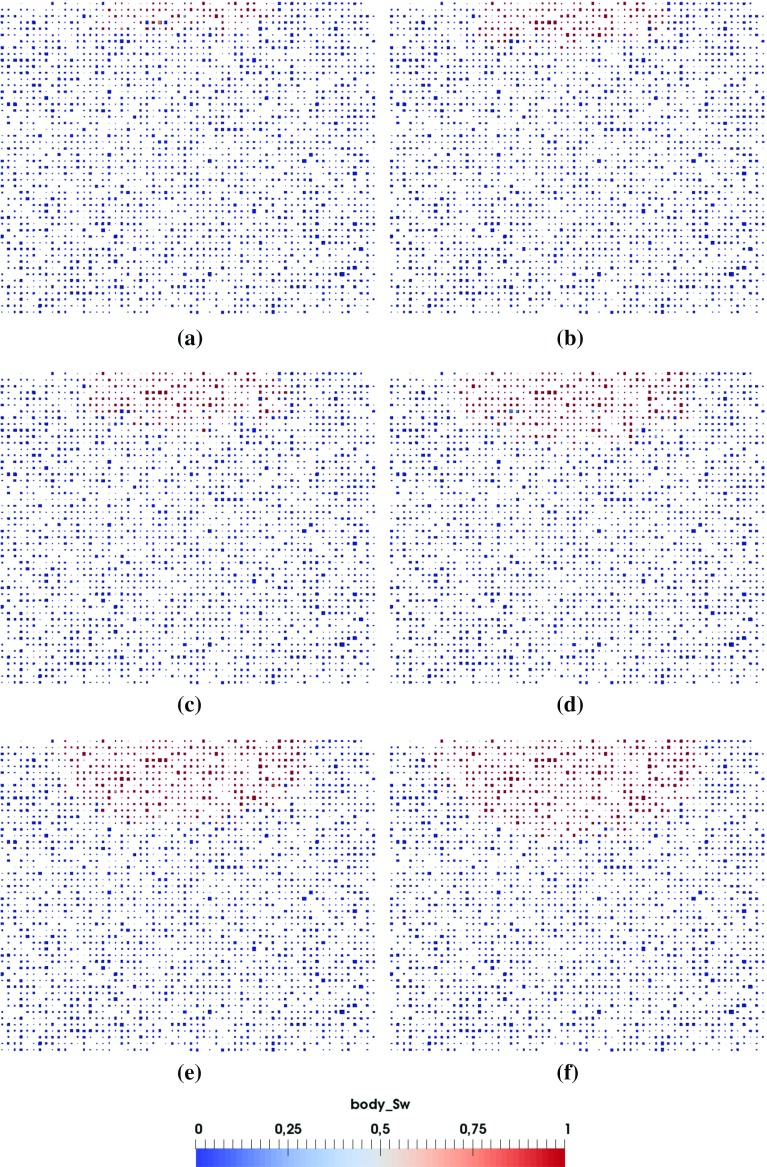
Cross-sectional view of the pore-scale saturation pattern evolution with time during the penetration of a 2 *pL* droplet (diameter of $16~\upmu \hbox {m}$, pore network G1, material properties M1). **a** $t=0.02~\hbox {ms}$, **b** $t=0.05~\hbox {ms}$, **c** $t=0.09~\hbox {ms}$, **d** $t=0.17~\hbox {ms}$, **e** $t=0.22~\hbox {ms}$, **f** $t=0.29~\hbox {ms}$

For a big droplet, the simulation of a big domain will be needed which would be extremely time consuming. One way to reduce the computational time is to simulate only part of the domain, considering the symmetric development of macroscopic saturation pattern. We will simulate a 2 *pL* droplet penetration first and check the symmetry of the resulting pore-scale saturation pattern. Based on the results, we determine the domain size of our simulations. The coordinate system is chosen so that the inlet is in the $x-y$ plane.

**Fig. 5:**
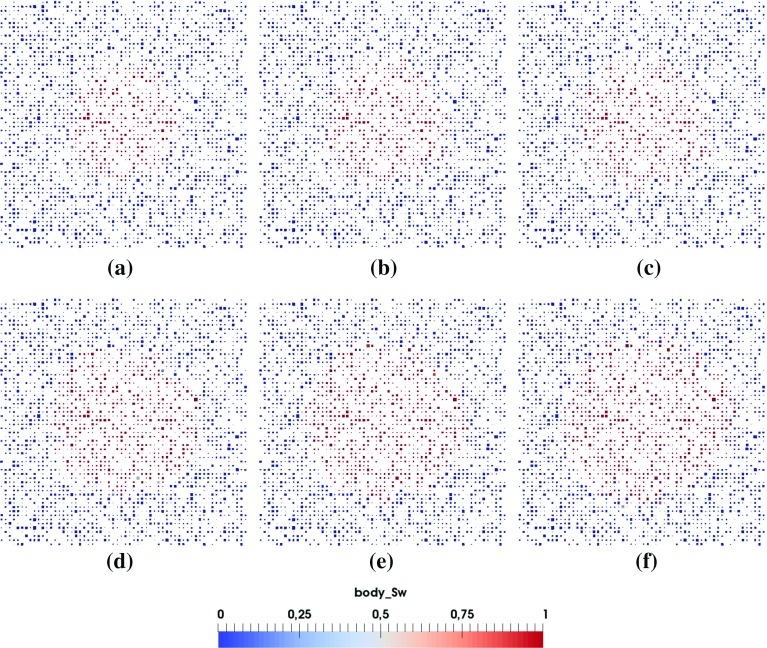
Top layer of the saturation pattern evolution from **a** to **f** during a 2 *pL* droplet penetration (diameter of $16~\upmu \hbox {m}$, pore network G1, material properties M1). **a** $t=0.02~\hbox {ms}$, **b** $t=0.05~\hbox {ms}$, **c** $t=0.09~\hbox {ms}$, **d** $t=0.17~\hbox {ms}$, **e** $t=0.22~\hbox {ms}$, **f** $t=0.29~\hbox {ms}$

Figures 4a–f and 5a–f provide the cross-sectional view and top layer of saturation pattern development, respectively, for a 2 *pL* droplet penetration into the full domain. As the saturation pattern develops, it becomes less symmetric due to the heterogeneity of the pore network. Nevertheless, Figs. 4 and 5 show that the main features of the process can be captured for simulation on 1/4 of the domain.

Therefore, in our dynamic simulations, we simulate 1/4 of the domain, namely penetration of 1/4 of a droplet into the porous medium. Domain size will be chosen large enough that no breakthrough happens at any confining faces of the domain. So, for simulation of droplets of different sizes, different domains sizes have been chosen. For simulation of 12 *pL* droplet imbibition, the network has 309470 pore bodies and 793852 pore throats. Only pressure in wetting phase saturated pore bodies are solved and simulation with (G1, M1) takes around 4 days on Intel Xeon(R) CPU E5-2667, 3.20 GHz.

### Penetration Time

#### Dynamics of Droplet Volume with Time

**Fig. 6:**
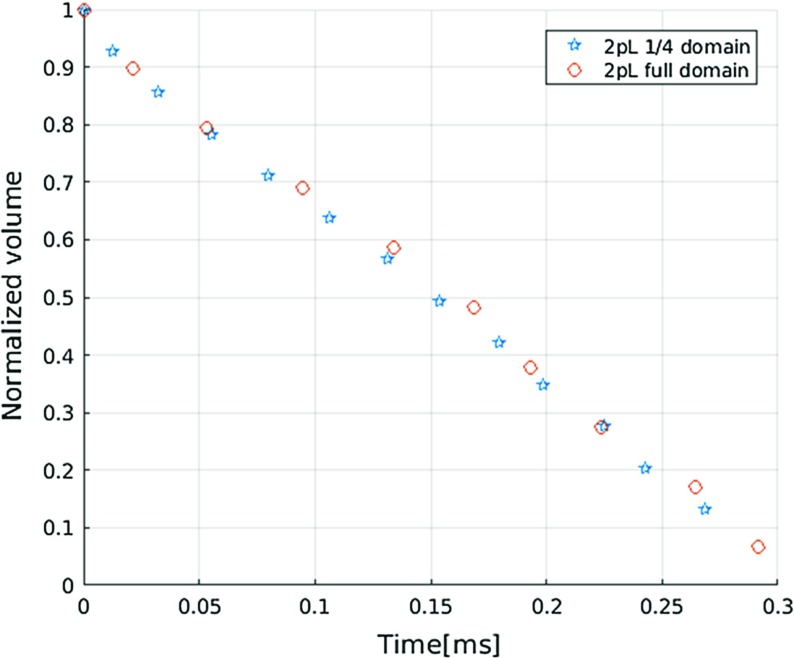
Variation of droplet volume as function of time for a 2 *pL* droplet in 1/4 domain and full domain (diameter of $16 \mu m$, pore network G1, material properties M1)

As a droplet imbibes into a porous medium, its volume decreases. Based on fluxes, we can calculate the change of its volume over time. Figure  6 shows the volume change as function of time for a 2 *pL* droplet obtained from simulations using two different domain sizes: 1/4 domain and full domain. Volumes are normalized to their initial values. Good agreement between these two simulations further confirms the adequacy of simulations for 1/4 of a droplet. In addition, we observe a linear decrease in droplet volume with time.

**Fig. 7:**
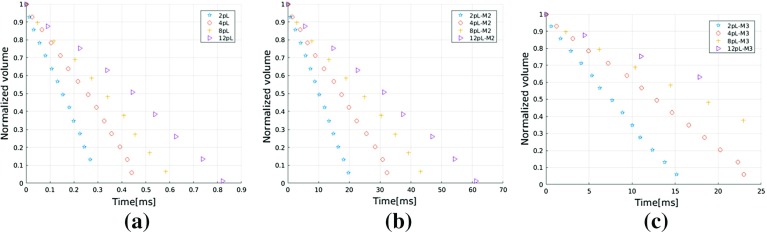
Variation of droplet volume as function of time for different material properties: **a** pore network G1 and material properties M1; **b** pore network G1 and material properties M2; **c** pore network G1 and material properties M3

Figures 7 shows droplet volume change with time for different droplet sizes and material properties. As expected, we can see that bigger droplets take longer to imbibe into the porous medium and lower interfacial tension, higher viscosity, higher contact angle decrease imbibition rate. Figure 7 suggests a linear decrease in droplets volume with time.

Thompson (2002) simulated liquid drop imbibition into fibrous material using two-pressure algorithm. Droplets were allowed to enter the network from a circle with equal diameter to the droplets themselves (no attempt to mimic the dynamics or hydrostatics of the drop outside of the network). For contact angle zero and 85 degree, a linear imbibed droplet volume over time was observed.

Using a picoliter spreading and absorption instrument, Clarke et al. (2002) found that a glycerol solution droplet took around 10–80 ms for about 90% of its initial volume to penetrate. Moreover, similar to our simulation, linear decrease in droplets volume with time was observed. Droplets in their experiments had a volume around 100 *pL* and four types of porous media with minimum pore sizes of 100, 220, 450, 650 nm were used. Interfacial tension was 0.038 kg $\hbox {s}^{-2}$ and wetting phase viscosity was 0.040 kg $\hbox {m}^{-1}\,\hbox {s}^{-1}$; the contact angle value was not reported.

These two findings together with our simulations, however, contradict the $\sqrt{t}$ trend which is obtained from macroscale simulations based on single-phase Darcy’s law. With the development of invasion pattern, on the one hand, wetting phase needs to overcome more viscous resistance, which could slow the imbibition process; on the other hand, more pores are accessible for liquid penetration. The nearly constant liquid imbibition rate should be the combined results of these two effects.

**Fig. 8:**
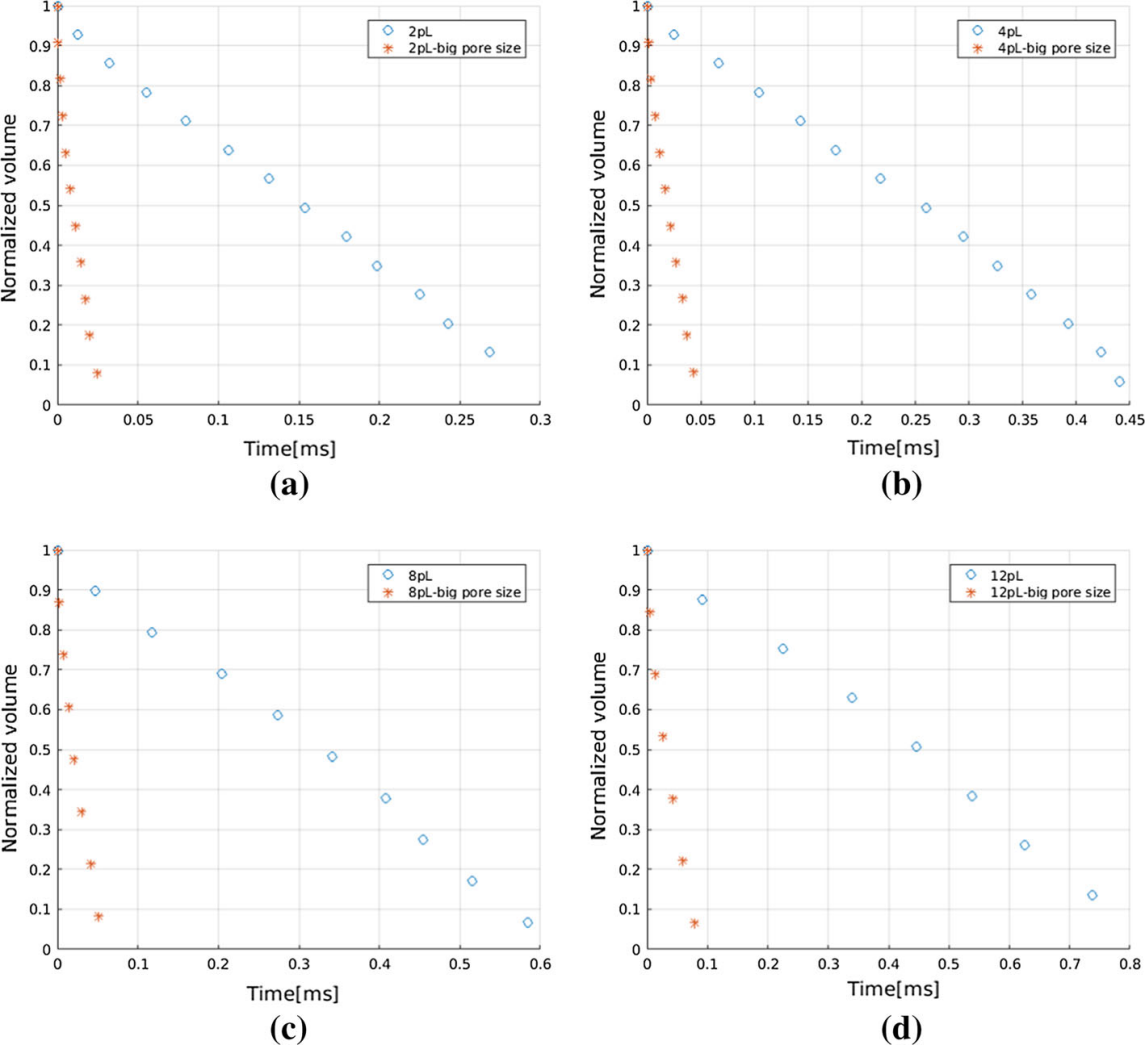
Effect of pore size on droplet volume change over time for droplets of different sizes using material properties M1: **a** 2 *pL* droplet; **b** 4 *pL* droplet; **c** 8 *pL* droplet; **d** 12 *pL* droplet

#### Effects of Pore Size on Penetration Time

We simulated penetration of different sized droplets into the pore networks G1 and G2 using material properties set M1. Figure 8 shows, as expected, how imbibition rate increases with increase in pore sizes. This is in agreement with experimental results in Clarke et al. (2002) and agrees well with Lucas–Washburn equation for a capillary tube. Moreover, we find a similar linear decrease in droplet volume with time for larger pore sizes.

**Fig. 9:**
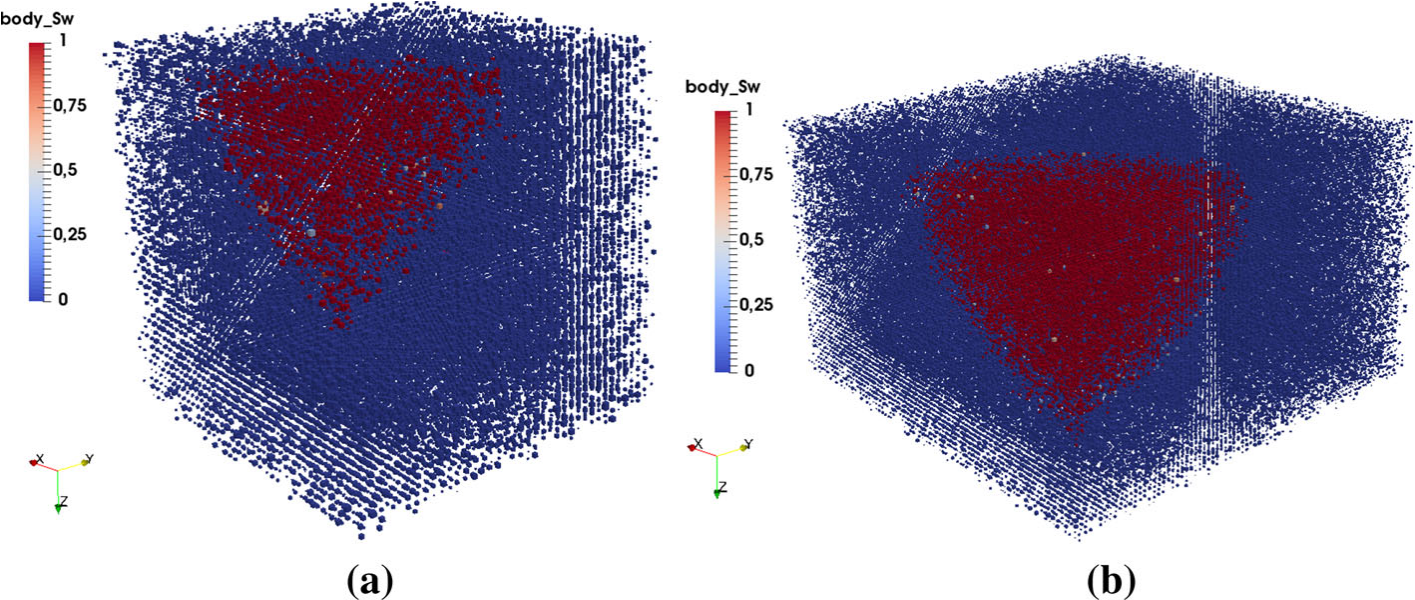
Saturation pattern just after a droplet has fully penetrated into porous medium (material properties M1). **a** 2 *pL* droplet, **b** 12 *pL* droplet

**Fig. 10:**
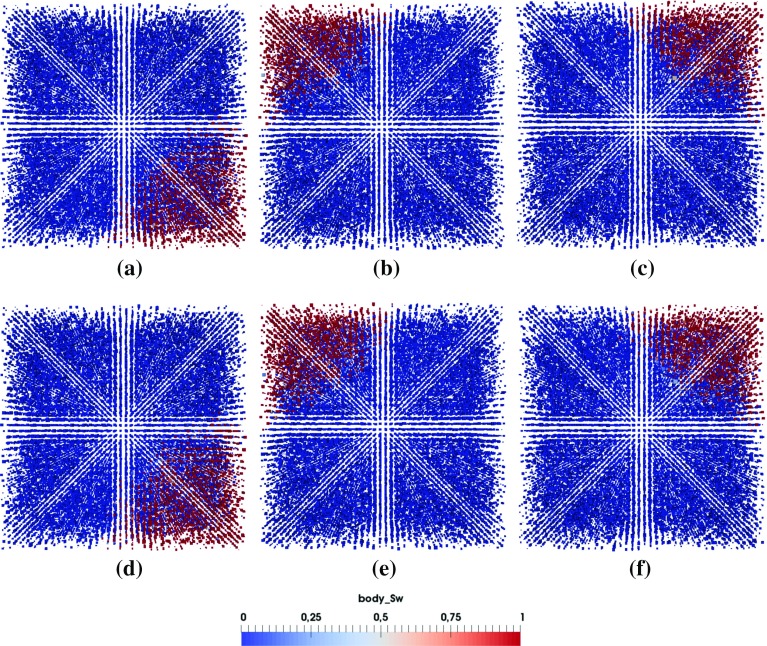
Top (*z*) and side (*x*, *y*) view of the saturation pattern of 2 *pL* droplet for different material properties (**a**–**c** M1; **d**–**f** M2)

### Invasion Pattern and Its Transition

In this section, we would like to explore whether a sharp invasion front exists during droplet penetration. We will check the invasion pattern just after droplet has fully penetrated into the porous medium. Simulations are performed on pore network G1 using material properties M1 and M2.

**Fig. 11:**
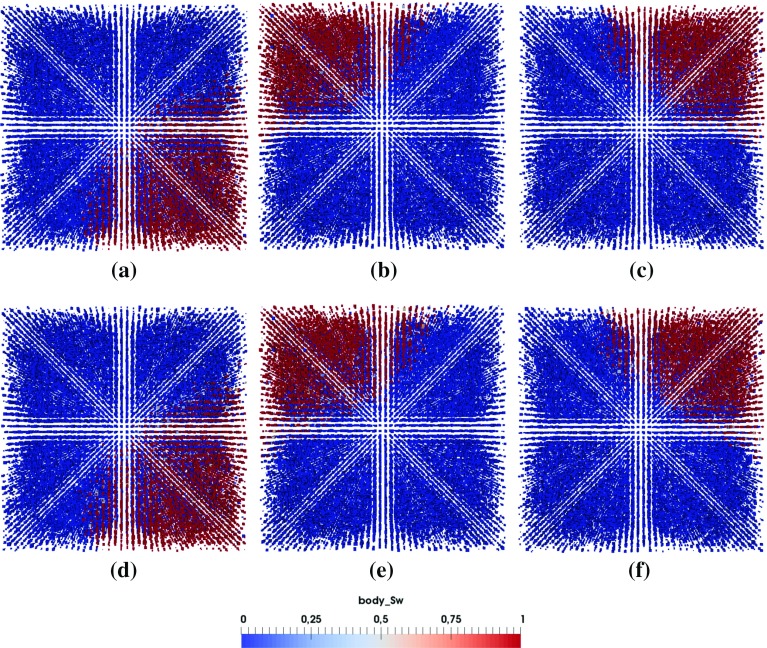
Top (*z*) and side (*x*, *y*) view of the saturation pattern of 4 *pL* droplet for different material properties (**a**–**c** M1; **d**–**f** M2)

**Fig. 12:**
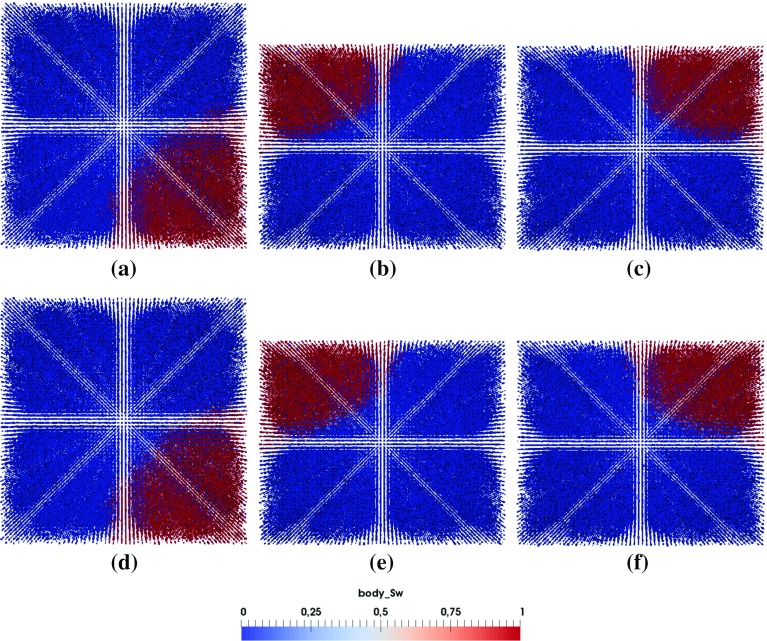
Top (*z*) and side (*x*, *y*) view of the saturation pattern of 8 *pL* droplet for different material properties (**a**–**c** M1; **d**–**f** M2)

Figure 9 provides a 3D view of the saturation patterns for droplets of 2 *pL* and 12 *pL* and Figs. 10, 11 and 12 show the top and side views of the saturation pattern for different droplet sizes with different material properties. For smaller droplet, saturation front is more sharper. Saturation pattern develops in all directions, so the problem is not fully 1-dimensional.

Due to the presence of small pore sizes together with pressure conditions at the boundaries, displacement is more piston-like and snap-off is suppressed as reported by Hughes and Blunt (2000). Still as we can see no sharp invasion front exists and the non-wetting phase stays behind the front. So to model liquid penetration in porous media at continuum-scale, Richards equation should be employed (Markicevic and Navaz 2010; Bear 1972). However, this requires knowledge of macroscopic properties, like capillary pressure-saturation relationship, relative permeability, which may be difficult to determine accurately.

**Fig. 13:**
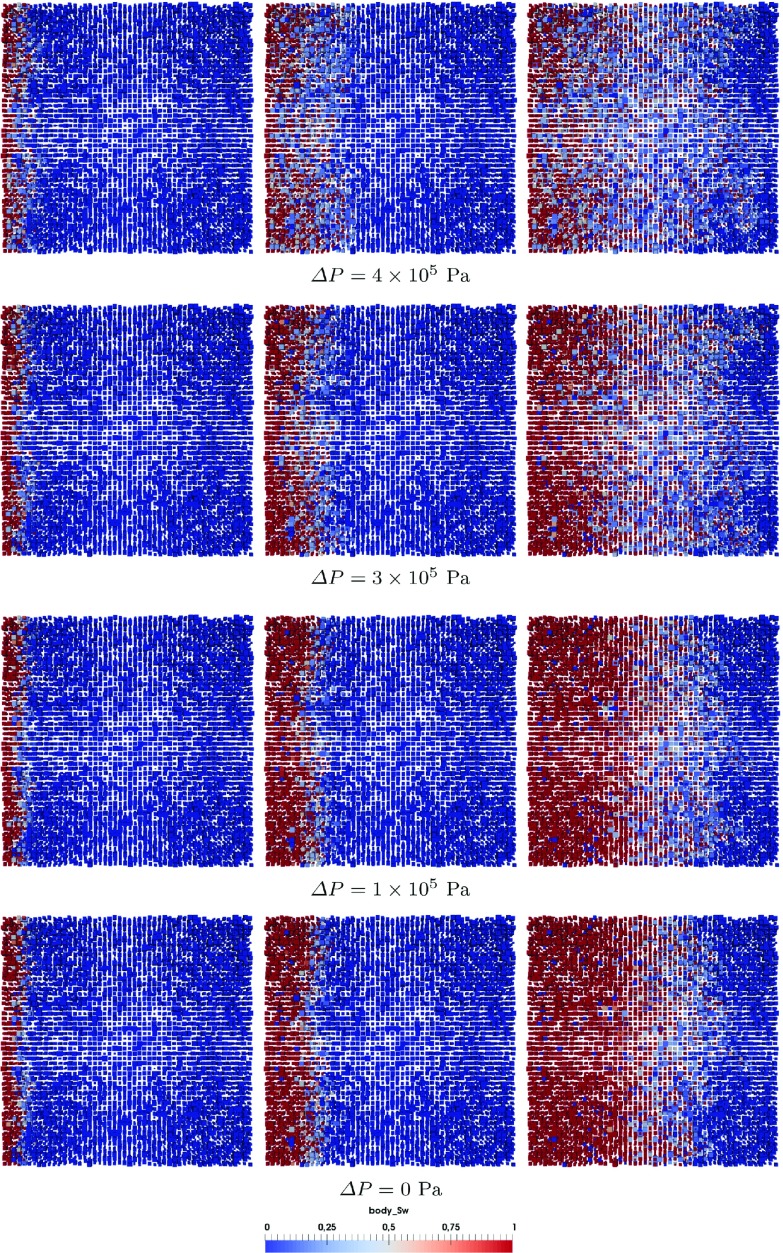
Top view of saturation pattern evolution with different boundary pressure difference $\Delta P= P_\mathrm{outlet}^{nw}-P_\mathrm{inlet}^{w}$

To check invasion front transition from a percolation-like pattern to a more sharper one, we have simulated imbibition in a pore-network with $50 \times 50 \times 5$ lattice using sizes distributions from Fig. 2. One side of the network is connected with wetting phase reservoir and the other side is connected with non-wetting phase reservoir. Fixed pressures are imposed at two reservoirs and no flow boundary conditions are set for side boundaries. Material property set M3 is used. Different pressure difference in non-wetting phase reservoir and wetting phase reservoir $\Delta P= P_\mathrm{outlet}^{nw}-P_\mathrm{inlet}^{w}$ are applied: $\Delta P = 4 \times 10^5$ Pa, $\Delta P = 3 \times 10^5$ Pa, $\Delta P = 1 \times 10^5$ Pa and $\Delta P = 0$ Pa (namely spontaneous imbibition).

Figure 13 provides top view of saturation pattern evolution for different boundary pressure difference $\Delta P$. As we can see for higher $\Delta P$, namely lower flow rate, saturation pattern is more percolation-like and there is more trapping of non-wetting phase in larger pores. As $\Delta P$ decreases, saturation pattern gets sharper with less trapping. No significant difference is observed for $\Delta P = 1 \times 10^5$ Pa and $\Delta P = 0$ Pa cases.

## Conclusion

In this work, we have developed a single-pressure dynamic pore-network model for primary imbibition and applied it for simulation of a droplet penetration into the coating layer of a coated paper. The model is based on an explicit-saturation implicit-pressure algorithm. However, it is made specially fast by solving pressure for fully wetting phase saturated pore bodies only. Geometry and topology of the pore network are based on FIB-SEM image of the coating material. Penetration of different sizes of droplets of different material properties into pore network of different geometries are performed. Droplet sizes are comparable with those of real inkjet droplets.

Simulations show a linear decrease in droplet volume with time, and this contradicts the expected $\sqrt{t}$-behavior in vertical imbibition that is obtained using macroscopic single-phase Darcy’s law. With the development of invasion pattern, on the one hand, wetting phase needs to overcome more viscous resistance, which could slow the imbibition process; on the other hand, more pores are accessible for liquid penetration. The constant imbibition rate should be the combined results of these two effects.

The examination of saturation patterns obtained from our simulations shows that no sharp invasion front exists and there is the presence of (residual) non-wetting phase behind the front. This means single-phase Darcy’s law is not appropriate for macroscale simulation of liquid penetration under these conditions. It is expected that Richards equation would be a better choice (Markicevic and Navaz 2010; Bear 1972), although one needs to deal with more complex properties, like capillary pressure-saturation relationship, relative permeability, which may be difficult to measure.

Our simulations also show transition of imbibition invasion front from percolation-like pattern to a more sharper one with less trapping of non-wetting phase as flow rate increases.

Future work is needed to incorporate the spreading of droplet on the surface of the porous substrate and couple the spreading and penetration process. Also, wetting phase behavior inside the porous medium after complete penetration of droplet should be studied. In this study, pure liquids have been the working fluids. Description of dye-based or pigment-based colorant movement should also be incorporated using more complex models.

## Figures and Tables

**Fig. 14:**
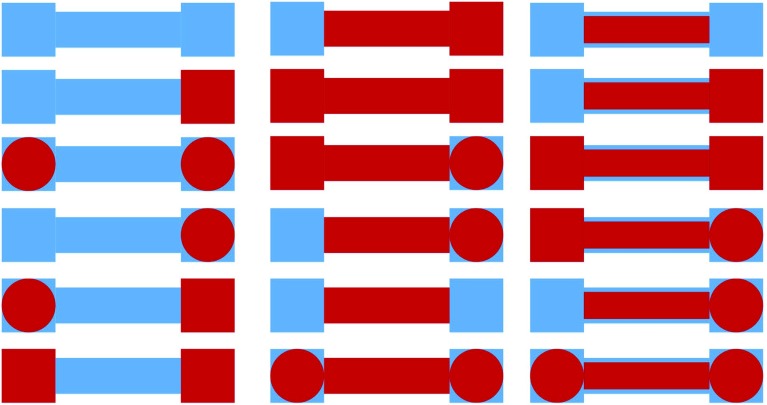
Possible liquid fillings within pore body and throats during imbibition

## Appendix: Possible Fluid Distribution Patterns During Primary Imbibition

There are 18 possible fluid occupancy of pores, shown in Fig. 14, where blue color shows the wetting phase and red color shows the non-wetting phase. Here, no attempt is made to represent the real interface shape. Due to assumptions in our primary imbibition algorithm, some of these configurations will not occur. We use A, B, C to denote three columns and 1–6 to denote the six rows. So we have 18 possible distribution patterns A1–C6 in Fig. 14. Below we explain which distribution pattern cannot be encountered and will be, therefore, excluded from considerations.

A2: As we do not consider volume of pore throats, so once there is wetting phase in the pore throat, neighboring pore body cannot be still fully saturated with non-wetting phase.
A5: See A2.
A6: It is not possible to trap wetting phase during primary imbibition in our algorithm.
C2: See A2.
C3: See A6.
C4: See A2.
